# Exploratory study of the underutilization of CTSA module services

**DOI:** 10.1017/cts.2022.440

**Published:** 2022-08-10

**Authors:** Julie T. Elworth, Melissa Vaught, Jillian Harvey, Rechelle Paranal, Adrienne Zell, Charbel El Bcheraoui

**Affiliations:** 1 Institute of Translational Health Sciences (ITHS), University of Washington, Seattle, WA, USA; 2 South Carolina Clinical & Translational Research Institute (SCTR), Medical University of South Carolina, Charleston, SC, USA; 3 Oregon Clinical & Translational Research Institute (OCTRI), Oregon Health & Science University, Portland, OR, USA; 4 Center for International Health Protection, Robert Koch Institute, Berlin, Germany

**Keywords:** service utilization, program evaluation, silo effect, CTSA, research services

## Abstract

**Background/Objective:**

The Clinical and Translational Science Award (CTSA) program aims to enhance the quality, efficiency, and impact of translation from discovery to interventions that improve human health. CTSA program hubs at medical research institutions across the United States develop and test innovative tools, methods, and processes, offering core resources and training for the clinical and translational research (CTR) workforce. Hubs have developed services across different domains, such as informatics and pilot studies, to provide *ad hoc* expertise and staffing for local research teams. Although these services can provide efficient, cost-effective ways to cover skills gaps and implement rigorous studies, three CTSAs of varying size found the majority of investigators were single domain service users, likely missing opportunities to further enhance their work.

**Methods:**

Through interviews with CTSA service users and a survey of CTSA service managers, this exploratory study aims to identify barriers to using services from multiple modules and solutions to overcome those barriers.

**Results:**

Barriers include challenges in finding information about services, unclear or unknown user needs, and users’ lack of funding to engage in services. More issues were identified for the largest CTSA.

**Conclusions:**

Although this study represents a small subset of CTSA hubs, we anticipate that our findings and proposed solutions will be relevant to the broader CTSA community. This study provides foundational information can use in their own efforts to increase service utilization and methods that can be used for more comprehensive studies that focus on explaining the relationship between CTSA features and rates of single versus cross-module service use.

## Introduction

In 2006, the National Institutes of Health established the Clinical and Translational Science Award (CTSA) Program to accelerate the process of moving research from discovery to delivery of treatments and diagnostics to patients [1]. The roughly 60 CTSA hub awardees aim to reduce the barriers to designing, initiating, and implementing clinical and translational research (CTR) studies through delivering a wide range of services, such as developing statistical plans, identifying prospective participants for research studies, and providing regulatory support for IRB and FDA submissions. CTSA services are provided through required modules (e.g., informatics, team science, and community engagement) and optional modules.

While some investigators, the “customers” of CTSA services, may only need services from a single module, many could benefit from engagement with multiple modules to achieve their CTR goals. Underutilization of CTSA services presents risks to the CTSA, investigators, and the CTSA’s home institution(s). For the CTSA hub, risks include missed opportunities for engaging investigators, unrealized income, fewer educational opportunities, and missed potential for overall impact. For the investigator, risks include unrealized rigor and inefficiency in study design, startup, and implementation, resulting in sub-optimal or delayed products. Rework and delays extend the length to completion, thus increasing costs and limiting staff and faculty effort that could be directed to other projects. These risks are likely elevated for early career investigators who lack resources and have limited experience with local institutional processes (e.g., IRB submissions). Taken together, these missed opportunities can reduce the value and impact of the clinical and translational funding portfolio at an institution.

Consequently, it became a source of concern when in 2018, the first author reported to the project investigator of their CTSA that during a 3-year period more than 75% of individuals (excluding REDCap users) who used CTSA services only engaged services from a single module. Given that this CTSA made available eleven modules offering a range of services, it seemed unlikely that so many investigators needed services from only a single module.

In 2019, the first author conducted preliminary interviews with seven investigators using services from multiple modules and drew on staff experience to gain early insights into service usage. (The 2019 Interview protocol is available in the Supplementary Material.) Both sources reiterated the value of CTSA services and the likelihood of investigators using services again. Four respondents felt that a central contact (e.g., a research navigator) was a useful conduit for forwarding them from one module to another, while two respondents found the services offered across modules on their own or through their colleagues. These preliminary data also highlighted barriers including: lack of follow-up by module staff; perceived high costs of service; lack of users’ awareness that services used were part of the CTSA; and lack of CTSA module staff knowledge of services offered by other modules. These early results suggested that, although many elements worked correctly for those who used services across modules, there were parts of the system did not work as expected.

Discussions with other CTSA evaluators sparked a collaboration between the Institute of Translational Health Sciences (ITHS) at the University of Washington, Oregon Clinical and Translational Research Institute (OCTRI) at Oregon Health and Science University (OHSU), and South Carolina Clinical & Translational Research Institute (SCTR) at Medical University of South Carolina (MUSC). The exploratory study presented here was selected by the Center for Leading Innovation & Collaboration (CLIC) at the University of Rochester as a Synergy Paper, a collaborative publication addressing challenges in clinical and translational research [2]. The key questions that this collaboration sought to address were:

- Is service underutilization observed at other CTSAs?
- Are there barriers to using more services across modules? If so, what are those barriers and what can be done to address them? Are other CTSAs experiencing similar problems?
- Are there elements of the CTSA that might support cross-module service use?

Through July 2021, CTSA budget limits were based on total institutional NIH direct costs (DC) awarded to the hub’s partners in the prior federal fiscal year, and CTSA hubs were stratified into three tiers on the basis of CTSA hub DC: small (<$4.5M DC), medium ($4.5–6M DC), and large (>$6M DC) [3]. This study included one CTSA of each size to provide insights into issues that might be related to hub size and/or complexity. The three participating CTSAs serve investigators across multiple organizations and, in some cases, entire states. Table 1 lists the selected characteristics of the three CTSAs.

**Table 1.** Selected characteristics of three participating Clinical and Translational Science Awards (CTSAs)

CTSA size (budget)	Small (<$4.5M)	Medium ($4.5–$6M)	Large (>$6M)
Year founded	2009	2006	2007
Organizational model	Single institution with collaborating organizations	Single institution with collaborating organizations	Three partner institutions with collaborating organizations
Geographic reach	One state	One state	Five states
Centralized Request System – Website	Yes	Yes	Yes
Navigator	Yes	Yes	Yes
Service fee structure	Majority of services provided at no-charge with some additional fee-based services	Some services provided at no-charge with additional fee-based services	Some services provided at no-charge with additional fee-based services

Our preliminary findings pointed to a potentially widespread and significant challenge across the CTSA. While there is extensive literature on service (under)utilization and customer (dis)engagement in business and health/social care settings, we anticipate that CTSA hubs—which focus on supporting research in non-commercial settings—may encounter different types of challenges and opportunities. In accordance with Babbie [4], we designed an exploratory study to better understand the issues at play, developed interview and survey questions, and tested the feasibility of an approach to investigate service underutilization at CTSAs.

## Material and Methods

Using data from preliminary interviews in 2019 and the first and last authors’ experiences, the same authors developed a root cause analysis (RCA) to define potential factors contributing to underutilization of CTSA services across multiple modules. The RCA served as a guide to the development of the interview protocol and survey instruments. RCA is an investigation technique increasingly used in healthcare and social sciences addressing health. While Andersen and Fagerhaug define RCA as a structured investigation that aims to identify the true cause of a problem and the actions necessary to eliminate it, this RCA was designed to provide an initial mapping of predicted causes of underutilization of CTSA services across modules [5]. Following data collection and analysis of the 2019 preliminary interviews, causal factors were charted and root causes identified. The initial charting was discussed and evaluated a second time by the first and last author based on the recurrence of the identified factors in the interviews. Some factors in this detailed RCA were included in the follow-up study, described below. An abbreviated version is illustrated as a fishbone diagram in the Discussion section. (See Supplementary Materials for the expanded version.)

### Service Utilization

Each participating CTSA hub maintains software for tracking hours of service provided for projects.^1,2^ We use the term “investigator” to refer to any individual using CTSA services, which may include faculty, research staff, or other contributors to research projects. Investigators who used at least 1 hour of service were pulled from each CTSAs’ respective databases. The list of investigators was sorted by the number of modules used in the time frame. Modules that solely provide funding for training and research (KL2, TL1, and Pilot) were excluded from the current study because awardees may have access to information about module services not afforded to other investigators.

In addition to services offered by modules, systems track the use of REDCap for electronic data capture, a primarily self-service data capture tool. For this reason, the standard use of REDCap was excluded from our analysis. However, substantive support, such as REDCap database development, was included as services under the relevant module.

### User Interviews

In the spring and summer of 2020, interviews were conducted with investigators at the small and large CTSAs to better understand investigators’ knowledge and experiences with the CTSA’s services; staff shortages at the medium CTSA precluded interviews there. Interview questions were similar to those used in interviews conducted in 2019. (The 2020 Interview protocol is available in the Supplementary Material).^3^ Those who had used services from a single module and those who used services across modules in calendar years 2018 and 2019 served as the population from which interviewees were selected, using two sample processes. At the large CTSA, investigators were sorted by frequency of module use. Investigators using services in a single module were sent up to two emails requesting their participation in the interview. If they did not respond or reported they could not participate, the next investigator listed was invited to participate. The same process was followed with investigators who used services from two or more modules, resulting in three investigators who used services from a single module and three who used services across two or more modules being interviewed at the large CTSA. At the small CTSA, a similar grouping process (one and two or more modules) was used, except investigators from each group were randomly selected for a total of three investigators who used services in a single module and two investigators who used services from two or more modules for a total of 11 completed interviews across both CTSAs. Originally, interviews with more investigators were planned; however the pandemic limited the availability of interviewees as many investigators were directly or indirectly working with COVID patients. Also due to the pandemic, all interviews were conducted via Zoom web conferencing software or over the phone using recording software. The modal category for the length of interviews was 11 min. Where investigators shared few experiences, interviews were brief, and where they shared more details about their experiences with services, the interviews were longer.

All interviews were recorded and transcribed verbatim. The general inductive approach, a common qualitative analysis method for program evaluation, was used to identify themes across the two CTSAs [6]. This method was adopted because of the contextual nature of service uptake within CTR was not fully understood. Authors from the small CTSA reviewed and summarized interview transcripts from both CTSAs and authors from the large CTSA reviewed transcripts and themes. Quotes that illustrated themes were highlighted.

### Survey of Module Managers

The survey of module managers at the three CTSAs, conducted in winter of 2021, was designed to explore features of modules that may be related to service use across CTSA modules. In comparison with faculty leads and other module staff, module managers were considered most knowledgeable about features of their CTSA modules, as they know who used services in their modules and the mechanics of providing those services.

Survey questions included those about the mechanics of referring investigators across modules, frequency of referrals, barriers to making referrals to and receiving referrals from other modules, whether the referral was a good match, and module managers’ confidence in their level of knowledge about other modules’ services. Module managers were also asked to identify the modules they worked in at the time of the survey, and for each module they worked in, the same set of questions was administered. (See Supplementary Materials for REDCap survey data dictionary.)

The survey was administered from ITHS and sent to the list of module managers submitted from ITHS, OCTRI, and SCTR. Similar to the interview methods, the KL2, TL1, and Pilot modules were not included in the survey. Team Science was mistakenly omitted from the survey for all three CTSAs. (See Supplementary Materials for the list of modules and the number of module managers responding.)

All 35 module managers were emailed a survey invitation via REDCap on February 12, 2021. Reminders were sent out weekly for 3 weeks to those who had not completed the survey. The survey closed on March 11, 2021. Across all three CTSAs, the response rate was 32 of 35 (Table 2). Most respondents worked in a single module, but some worked in two or more. The survey data were downloaded from REDCap and analyzed with SPSS and Excel.

**Table 2.** Clinical and Translational Science Award (CTSA) module manager survey response rates

CTSA	Small	Medium	Large	Total
Response rate (responding/total invited)	10/11	6/7	16/17	32/35
1. Module	8	5	11	24
2. Modules	2	1	2	5
3. Modules	0	0	2	2
4. Modules	0	0	1	1
Total	10	6	16	32

## Results

Leveraging service tracking data from each CTSA, we calculated the number and percentage of investigators who used services from a single module during a 36-month period (Table 3). The functional and organizational structure of services offered by each CTSA vary substantially, so the interpretation of the volume of use is limited. For example, at the medium CTSA, biostatistical services are provided through a university core, and thus not part of the CTSA’s service/investigator count. However, each CTSA has 7 to 11 modules offering services. Across the three CTSAs, more than half of investigators only used services in a single module, with the large CTSA having the highest proportion of single module users.

**Table 3.** Investigator use of Clinical and Translational Science Award (CTSAs) module services

CTSA	Small	Medium	Large
Date range	01/01/2016–12/31/18	01/01/2016–12/31/18^a^	06/01/2015–05/31/2018^b^
Unique investigators^c^	1240	350	1130
Number (percentage) of investigators who used services in a single module^c^	721 (58.1%)	214 (61.1%)	892 (78.9%)

a This date range includes a CTSA funding gap year.

b This date range was retained for consistency with that used in the grant application.

c All investigator numbers exclude REDCap users.

Using qualitative responses from investigator interviews conducted in 2020 and quantitative and qualitative responses from the 2021 survey of module managers, we defined four categories of issues that could affect service utilization: *Initiation* of services, *referrals* across modules, *resources* available to investigators, and *services* or module-specific features. Each of these categories is discussed below.

### Initiation

Interviews with users of CTSA services suggested that it can be challenging for investigators to identify the services offered by the CTSA. CTSA websites may be challenging to use or lack information that investigators seek. One respondent observed, “*I find it’s really hard to figure out exactly what services they have… And then when even trying to figure out information on your own through a website, the website is missing a lot of detail as well.*” Another reported a counter-intuitive experience where they were asked to provide information related to the very issue that initiated the request, *“…the only thing that I found a little bit difficult was some of the questions that I was required to answer were the reasons that I wanted to set up the meeting in the first place*.” Among those offering the services, one module manager reported in the survey that keeping all the services in mind was difficult, suggesting that some module managers may find it challenging to direct investigators to the correct service.

Another challenge reported by investigators was the absence of a follow-up once a request was submitted. One investigator said, *“Yeah, they need to have a knowledgeable central source that you can contact quickly… emailing and then waiting for days to get a response is not conducive to doing research quickly.”* Another said, *“…I was just sort of waiting for a response, but it was not in the system, so it was just not clear…what was going on.”*

### Referrals

There are mixed results about CTSA referral services. Interviews with some investigators who had previously used a CTSA service revealed that, for some, there was no apparent effort to follow-up with them about other CTSA services they might need. One respondent said, *“No one from [CTSA] has ever contacted me and said, ‘we know you use the [CTSA]…here are these other services and would you like to use those.’”* Yet another investigator reported experiencing some confusion about which part of the CTSA or even the university they were dealing with:

*“… I was placed in contact with different people…it was a bit confusing. So, it’s not under the same umbrella…that’s my understanding, they each had a different role or jobs in different organizations or institute or university. So, that’s a bit confusing.”*

In contrast, another investigator reported, *“So, I had made a request for a [service] and it was not approved, but at that time they suggested a different route of [CTSA] funding for me, which was super helpful.”*

Though the majority of module managers reported no barriers to make referrals, one module manager wrote, *“When referring clients to other modules, I rarely know if the client reached out to the other module or* (if) *module staff followed up with the client.”* Another module manager reported, *“I’m not always a good judge about whether a particular client is a good fit for another [module].”*

Among the nine of 32 module managers who reported barriers (Table 4), the two cited by module managers in all three CTSA were *clients’ needs are unknown or unclear* and *clients’ lack of funding.* The large CTSA had the most barriers identified by one or more module managers.

**Table 4.** Barriers to service use by Clinical and Translational Science Award (CTSA) size

CTSA	Small	Medium	Large
Client’s needs are unknown or unclear	✓	✓	✓
Client lacks funding to use services in other modules	✓	✓	✓
Lack of time to make a referral to another module	0	✓	✓
Other modules do not have the resources to accept more clients	0	✓	✓
Client refusal or delay	0	0	✓
Lack of knowledge of the services offered by other modules in the CTSA	0	0	✓
No process for referring clients to other modules	0	0	✓
No professional relationship with other modules	0	0	✓
Lack of comfort referring clients to other modules	0	0	✓
Other: limitations on part of investigators	0	0	✓

**Fig. 1.**
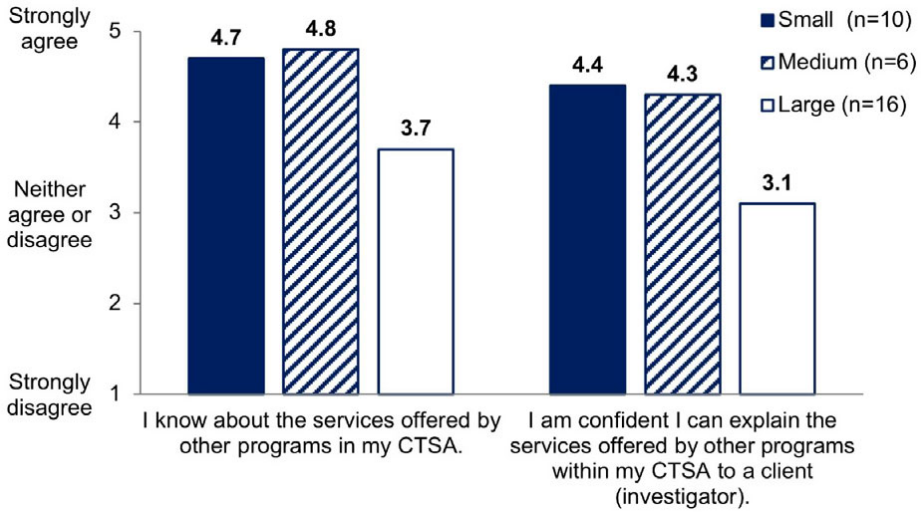
Level of agreement about knowledge and confidence by module managers by Clinical and Translational Science Award (CTSA) size.

When asked their level of agreement with the statement “*I know about the services offered by other [modules] in my CTSA*,” over 80% of module managers reported they agreed or strongly agreed. A smaller percentage, just under 72%, agreed or strongly agreed with the statement, “*I am confident I can explain the services offered by other [modules] within my CTSA to a client*.” Figure 1 shows the average level of agreement by the size of CTSA funding. Module managers responding to the survey from the small and medium CTSAs have much higher levels of agreement, with means at or above 4.3, compared to both the knowledge and confidence statements of the large CTSA with means at or below 3.7. This suggests a possible relationship between CTSA size or capacity and the level of knowledge or confidence in the knowledge of other modules, expressed by managers. A module manager from the large CTSA reported, *“I’m only aware of referrals that go to other [modules] in which I work…”* A longer tenure at the CTSA can facilitate knowledge of other module services. A module manager reported that their years of experience and personal contacts with managers in other modules within their CTSA were the primary source of information about services in those other modules.

Module managers were asked to report the frequency of referrals from their module to other modules that resulted in a “good” match (Table 5). Fifteen managers reported that matches were *often* good ones, followed by eight managers who felt that matches were *always* good ones. Five were *not sure* if the matches were good, while one each thought matches were *sometimes* and *never* good ones. Four out of the five managers who reported that they were “not sure” of the frequency of good matches were from the large CTSA.

**Table 5.** Frequency with which referrals have been a good match from the respondent’s module to other modules

Clinical and Translational Science Award (CTSA)^a^	Small^b,c^	Medium	Large
Biomedical informatics	–	–	5
Community engagement	3	3	3,5
Translational workforce development	–	5	3,4
Biostatics, epidemiology, and research design	4	4	3,5
Participant and clinical interactions	4	4	3,3
Regulatory knowledge and support	4	1	3,3,3
Integrating special populations	3	–	–
Liaison to trial innovation centers	4		3,3,3,5
Liaison to recruitment innovation centers	4		2
Gene and cell therapy lab			3
Mode	4	4	3

Numbers in the table are the individual scores from module managers (1 = never; 2 = sometimes; 3 = often; 4 = always; 5 = not sure).

a Only respondents who answered in a preceding question that they referred investigators from their module to one or more other modules were asked this question.

b Dashes indicate no module managers answered this question.

c Blank cells indicate the module is not offered at this site.

The results are similar for the frequency of good matches referred *from* other modules. The modal category for the frequency with which the referral to the respondents’ modules *from* other modules was good matches was “often” with 14. Seven reported that matches were “always” good, and one reported that “sometimes” matches were good (Table 6). CTSA size appears to be less of a factor in referrals from other modules to the respondents’ modules.

**Table 6.** Frequency with which referrals have been a good match from other modules to the respondent’s modules

Clinical and Translational Science Award (CTSA)^a^	Small^b,c^	Medium	Large
Biomedical informatics	–	–	3
Community engagement	3	3	2,3
Translational workforce development	4	–	3
Biostatics, epidemiology, and research design	4	4	4
Participant and clinical interactions	3	4	3,3
Regulatory knowledge and support	3	3	3
Integrating special populations	3	–	–
Liaison to trial innovation centers	–		3,4
Liaison to recruitment innovation centers	3		4
Gene and cell therapy lab			–
Mode	3	3 & 4	3

Numbers in the table are the individual scores from module managers (1 = never; 2 = sometimes; 3 = often; 4 = always; 5 = not sure).

a Only respondents who answered in a preceding question that they referred investigators from their module to one or more other modules were asked this question.

b Dashes indicate no module managers answered this question.

c Blank cells indicate that the module is not offered at this site.

### Resources

Resources, including the cost or perceived cost of module services and the availability of services from non-CTSA centers, were a third source of underutilization. For some investigators, the lack of funding precludes the use of more CTSA services. One investigator reported, *“Cost is always a consideration…I would have used more services if I had more funding for some things.”* Another investigator stated simply, *“It was very expensive.”* A module manager said, *“Some unfunded/underfunded studies don’t have the $ to pay for services or workload doesn’t allow for the assistance the client needs on the timeline they need it.”* In contrast, another study investigator warned about the risk of avoiding CTSA services because of cost:

*“I feel that when you are penny wise and pound foolish that I think people tend to look at the smaller issues and they say, ‘wow, you know, I can get this done for this price’. And yet when you’re talking about losing valuable samples or not having a place that’s centrally located for your participants…”*

The data also suggested that some services duplicated those offered by other parts of the same institution. One investigator observed, *“Now I know that pediatrics has that [service] and it’s kind of competing.”*

### Service

Another area expected to contribute to underutilization is *service*, specifically a lack of specific expertise, capacity, or both. Results from the interviews with investigators suggested that some service providers may not be familiar with the “real-world” context of the investigators’ research and, as one investigator put it, that ability “…*is so important for the success of the project*.” The service provider may also be unfamiliar with particular terminology or other elements of a project. One investigator observed, *“….we had delays or needed to check in about something. It was because, like, they’re just not used to the medical terminology that is necessary to do the data abstraction.*”

In other instances, an investigator may request services from one module, but module staff may realize that services from another module are a better match for the investigator’s needs. In this case, staff representing multiple modules may be brought together to consult on a project. As one module manager wrote, *“….a researcher may put in a request for [Community Engagement], but after discussion we realize it may be advantageous for [other modules] to be present for the discussion.”* This, of course, requires that module staff know the services provided by other modules and that procedures are in place for joint consults. Limited capacity to accept more clients is a factor at the large and medium CTSAs, according to module managers at those sites.

An issue expected but not reported in responses was that investigator expectations do not match the quality of the service delivered. During the interviews, most investigators report being satisfied with the services received. One investigator observed, “*I found the [service] extremely helpful*.”

## Discussion

**Fig. 2.**
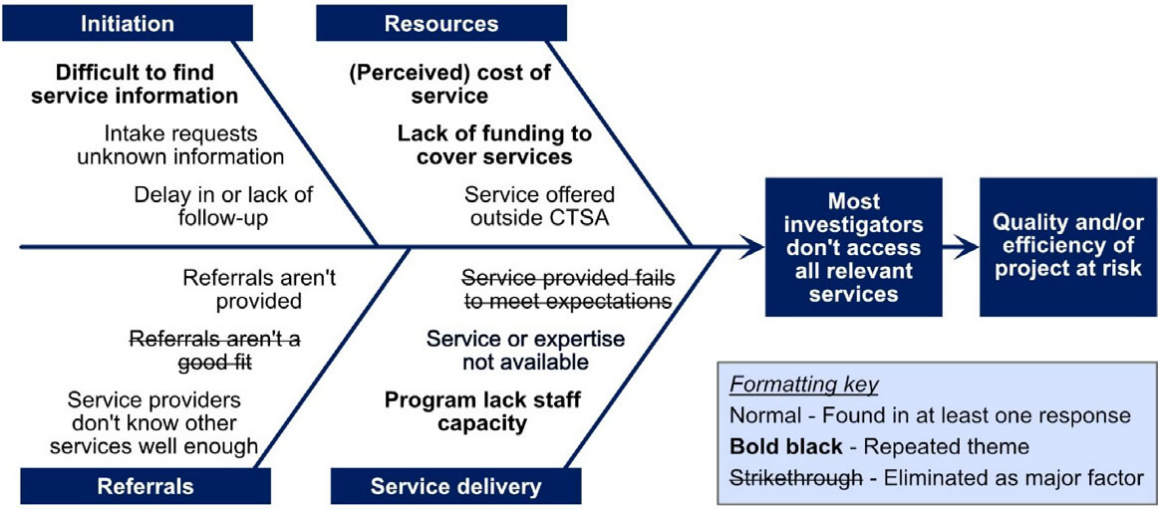
Factors expected to contribute to the underutilization of services across Clinical and Translational Science Award (CTSA) Modules.

This study began with the observation that at three CTSAs, a large majority of investigators (58% to 79%) only use services from a single CTSA module. Figure 2 summarizes factors that could contribute to this phenomenon and that we could assess through our interviews and survey. As an exploratory study, these findings may not extrapolate across the CTSA consortium. For example, we do not know how widespread service underutilization is or which, if any, CTSA factors contribute to underutilization. On the other hand, other CTSAs have shared anecdotal evidence of similar challenges in response to our presentations on this topic. Accordingly, our results support recommendations for solving key barriers in each category of issues identified.

Finding and requesting services can be improved. First, organizations should ensure that the CTSA website is well-constructed, clear, and easy for CTSA stakeholders to use, including newcomers to the CTSA. CTSAs could consider including a “Start here” button to orient new users of module services, trainings, and award opportunities. In addition, CTSAs could also consider creating and posting overview webinars of module services, including the kinds of questions answered by each of those services, how to access services, costs, and questions that service providers will be asking investigators at their first meeting. Webinars would need to be reviewed regularly to ensure that information is current about module services. Resources and other materials that help clarify elements of these services can be added and accessed via links posted on the CTSA website.

Intake forms should omit asking detailed questions. Questions on intake forms should be limited to those that ensure that the service provided matches the needs of the investigator. Where project information does not match services supplied, a mechanism should be added that directs the investigator to the correct module. A central contact across all module services (such as a research navigator) is an especially useful role in CTSAs. The navigator matches investigators’ project needs with the relevant modules and can follow-up when additional services are needed.

Referrals from services in one module to those in another need special attention in the form of a referral management system where, immediately following delivery of a service there is a formal process that includes identifying key next steps for the investigator in their project, and then the services needed to achieve those next steps. Optimally, this referral process includes a formal hand-off from one module followed by a formal reception from staff at the next module. The hand-off and reception should be conducted in a reasonable length of time to ensure that the investigator knows that they have not been forgotten. It may be helpful to provide new users with a brief introduction to the structure of the CTSA hub and services. CTSAs integrate resources and personnel from across their partner institutions, and many are multi-institutional collaborations. Investigators may be confused if they are provided with a referral to what appears to be a different institution or center that partners with the CTSA to offer selected services.

CTSA modules may also need to consider ways to better co-create and communicate service offerings and value for investigators. Service-dominant logic considers how connections between individuals, organizations, and networks can integrate and apply specialized skills and knowledge for the benefit of customers and partners [7]. A central premise of service-dominant logic is that the provider can only make a value proposition; services lack value until used, and the value is co-created and determined by the customer when a service offering is applied to their needs. In some cases, modules are the sole provider of certain key services at their institutions (*e.g.,* extracting data from the enterprise data warehouse). Such cases provide a clear value proposition with no substantive competition. In other domains, modules fulfill tasks that could be served by a member of the investigator’s team or a new hire, but CTSA service providers offer flexible staffing and bring institutional and regulatory knowledge and experience. Other services may deliver the most value when deployed upstream from where investigators may consider them, for instance, designing for implementation at early stages of intervention development rather than waiting until the clinical stages of CTR. As noted in an interview, investigators may focus primarily on “hard” dollars—the direct cost savings and/or return on investment of a service or product. CTSA services may appear more expensive than alternative options in terms of “hard” dollars but deliver “soft” dollar savings—e.g., fewer hours or resources needed to complete a task due to higher efficiency, improved rigor and reproducibility, and/or reduced rework. Communicating these benefits may overcome some barriers to CTSA service use.

Resource barriers can be addressed by CTSAs by providing clear information on all service costs, including information on services provided at no cost to investigators, the conditions of those services, what services or parts of services are subsidized, and which are charged full costs. Clarification of assumptions and estimated length of time for service completion should also be supplied to investigators before services are provided. Investigators should be given clear information and guidance about budgeting for services, for example, in a grant application. Also, any additional funding options should be shared with investigators, for example, some CTSAs provide in-kind service vouchers for early-stage investigators.

CTSA module teams need to calculate their capacity to provide each of their services and adjust intake accordingly. If demand for services exceeds capacity, the module team needs to either expand capacity or alert investigators of the anticipated length of delay. Approaches to increasing efficiency including self-service tools and cross-functional staffing should also be considered.

Other barriers to service can be addressed by improving communication across modules. For example, the hub could maintain a current list of services offered and in which modules those services are available, as well as those services that have been retired. In addition, there may be services that are not covered but should be, or that are not recognized as relevant by investigators. Preclinical researchers conducting in vitro and animal studies, for example, may only leverage biostatistical support. However, preclinical projects benefit from early end-user feedback, provided for instance through Community Engagement collaborations. Alternatively, there may be the same or similar services offered by more than one module. Depending on the needs of investigators served by the hub, this overlap may be attractive. A “map” of these services by module, including overlap, retired, and missing services should be updated at least annually and reviewed by directors and executive leadership of the CTSAs for strategic planning.

The size of CTSAs may influence the effectiveness of information exchange and referrals. In this study, the small and medium CTSAs have one institution each, whereas the large CTSA covers three partner institutions located in the same city but physically dispersed. Decentralization likely reduces opportunities to communicate and share information across modules. Module manager self-efficacy ratings relating to other modules (shown in Fig. 1) suggests that greater organizational complexity and/or geographic dispersion of the CTSA impedes communication across modules. This issue may have become more common among CTSAs, regardless of size and complexity, now that more teams work in dispersed environments as a result of the COVID-19 pandemic. Tactics to facilitate regular communication across modules should be considered, such as regularly scheduled meetings to discuss services, changes in services, problems with referrals, and capacity issues. Meetings and other communications should also include navigators, who need to stay abreast of services offered and retired.

The survey data on the frequency of “good” matches tended to be higher when the managers responding to the question *made* the referral versus *received* referrals from other managers and may be a reflection of elevated self-confidence versus confidence in others to make successful matches. Nevertheless, “good” matches made by the respondents were more frequently reported for the two smaller CTSAs than the larger CTSA, suggesting that size and/or complexity may be an important variable explaining differences in successful matching across modules.

### Future Research

In our explorations examining this phenomenon, we referenced different models focused on service delivery, consumer preferences, and service uptake. Life-cycle models address areas of temporal decision-making where current factors, such as actual or perceived available resources, influence the allocation of resources and thus create or limit the next set of options available [8,9]. Utilization management models focus on decisions and processes that manage healthcare costs on an individual case basis to reduce waste and unnecessary service and product use while also identifying cost-effective and necessary interventions that provide the most effective outcome (quality) [10,11]. Socio-cultural models took into consideration the highly complex institutional and environmental norms along with the interpersonal perceptions embedded within an enterprise and how these dynamics impact adoption [12–16]. We found that these models were incomplete in addressing our specific inquiry regarding research support services and their use. The clinical and the CTR environments appear to be similar, and at times even share infrastructure, so there may be a presumption that models applied in a clinical setting could easily be extrapolated to the CTR environment. However, these arenas prove to have very different starting points when it comes to consumer entry to services. For instance, in a clinical setting that considers socio-cultural influences for service uptake, it needs to account for cost-recovery, medical literacy, and other factors associated with social determinants. For a CTR environment, those factors tend not to come into play until much later in the process and that the CTR starting point for utilization would actually occur earlier, for example, when determining study participant recruitment feasibility.

Additionally, CTSA organizational structures may be uniquely complex. The objectives of CTSA hubs span many disciplines, drawing on expertise across numerous divisions and departments. CTSAs typically partner and provide services across multiple enterprises that operate independently of one another, for example, connecting a university and its affiliated healthcare system, an independent pediatric hospital, community health systems, and/or independent research centers. Though this broad engagement within and between institutions enables hubs to develop innovative and generalizable advances in translational science, it also produces silos. Decentralization has potential benefits, as teams can become very familiar with needs and resources in their domains, but silos can create or amplify challenges with communication and collaboration [17]. For example, insufficient communication and cooperation across silos can limit opportunities to leverage successes within the organization and to cross-promote relevant services and resources. Other risks of silos include focusing on the work within the silo at the expense of the organization, a lack of flexibility, and losing sight of “customer” needs [18].

Beyond silos, there are numerous other factors that may limit the use of available services. Service quality is variable, depending in part on the provider, and due to many intangible and inconsistent features, a customer’s evaluation is more challenging for services than for goods or tools [19]. Furthermore, services offered by CTSAs are generally more functional and utilitarian (vs. participative and co-creative), and functional services tend towards transactional relationships and higher customer disengagement once objectives are met [20]. Future research should consider these issues as they develop models to explain outcomes.

Our study has certain limitations that can be addressed in future research. First, we used an RCA as a basis for the development of our interview guides and surveys. RCA, like most qualitative data analysis methods, can be challenged by previous assumptions or researchers’ preferences. However, this study addresses topics that are fairly insensitive, one of the reasons why RCA is increasingly recommended as an analysis tool for its utility in program evaluation [21]. Second, with this small sample of investigators and CTSAs, we cannot generalize either to the populations of investigators using any number of module services or to the population of CTSAs. Third, investigators who agreed to interviews are possibly a biased sample of all investigators in the population of users from each of the three CTSAs. For example, investigators who are especially busy may experience other barriers to service use than those interviewed in this study. Fourth, the number of module managers responding to the survey from each CTSA could be the source of the different counts of barriers rather than the characteristics of the CTSAs.

The findings of this exploratory study warrant more comprehensive studies that include a much larger number of CTSAs and that are able to adapt relevant models to explain outcomes. Such studies could answer questions about the pervasiveness of the underutilization problem including the severity of the problem and how that problem is exhibited across types of CTSAs. For example, are there other CTSAs with similar distributions of use across modules? If so, what are the characteristics of those modules and how do those characteristics contribute to the distribution of module use? Are there CTSAs that have a higher rate of cross-module use? If so, how do the features of their CTSAs compare with the features of CTSAs with dominant single module use? How do formal and informal networks operate where cross-module service use is high versus low? Finally, what is the relationship between the use of services across modules and quality, count, and speed of products? Answers to these questions will help with understanding the extent and impact of underutilization, with the tailoring of solutions to CTSA type and conditions, thereby facilitating the mission of the CTSA.

## Footnotes

1 All three sites have conducted needs assessments used to adapt modules and to provide and tailor services to the users of their CTSA services.

2 The small CTSA used SPARCRequest to store their service user data; the medium CTSA used an internal system that ran off of Salesforce; and the large CTSA used an internal system, RISE.

3 The large CTSA received a formal IRB exemption (Category 2), and the small CTSA considered the interviews conducted under quality improvement and not subject to IRB review.

## Supplementary material

For supplementary material accompanying this paper visit https://doi.org/10.1017/cts.2022.440. click here to view supplementary material
