# Supplementary material for: Exploratory study of the underutilization of CTSA module services
Source: J Clin Transl Sci. 2022 Aug 10;6(1):e114. doi: 10.1017/cts.2022.440 (PMC9549576; doi:10.1017/cts.2022.440)
Supplement: Supplementary file 1 [file S205986612200440Xsup001.zip › S205986612200440Xsup001.docx]

**2019 Interview Protocol**

**Goal:** The goal of the interview is to glean experiences of those who used services from more than one or two ITHS programs, between June 2015 and December 2018.

**Introduction:** The University of Washington’s Institute of Translational Health Sciences or ITHS has provided services to medical researchers for about 11 years. At ITHS we’re interested in understanding the way our services are used and we’re interviewing some investigators who have used two or more ITHS services in the last 3½ years. The interview is voluntary. It is neither confidential nor anonymous, but I won’t be divulging your identity unless it is important, for example, there is something that our PI needs to follow-up on. Do I have your permission to record this interview? I’ll use the recording to augment my notes.

Q1. When was the last time you remember using ITHS services?

What service or services did you use then?

How did that go for you?

Q2. I’d like to take a step back and ask how you found out about ITHS services.

Were there multiple sources of information or just one?

Who was that source (or where those sources)?

Q3. What are the other services have you used from ITHS? (Check match in answers to Q4)

Q4. It looks like you used (a-d filled in prior to interview)

___ a.____________________ for project _________________ in (date) _____________

___ b.____________________ for project _________________ in (date) _____________

___ c.____________________ for project _________________ in (date) _____________

___ d.____________________ for project _________________ in (date) _____________

Q5. Did you come back to ITHS each time or was there someone who guided you to the next service?

Q6. Did anyone follow-up with you about other services you might need? (E.g. writing a grant, sample selection, statistical help, analysis, publication)

Q7. Do you think you would use any of those services again?

Q8. Do you think your colleagues would benefit from any of these services?

Q9. Do you have any comments you would like me to add to my notes?
