# Supplementary material for: Exploratory study of the underutilization of CTSA module services
Source: J Clin Transl Sci. 2022 Aug 10;6(1):e114. doi: 10.1017/cts.2022.440 (PMC9549576; doi:10.1017/cts.2022.440)
Supplement: Supplementary file 1 [file S205986612200440Xsup001.zip › S205986612200440Xsup002.pptx]

## Slide 1
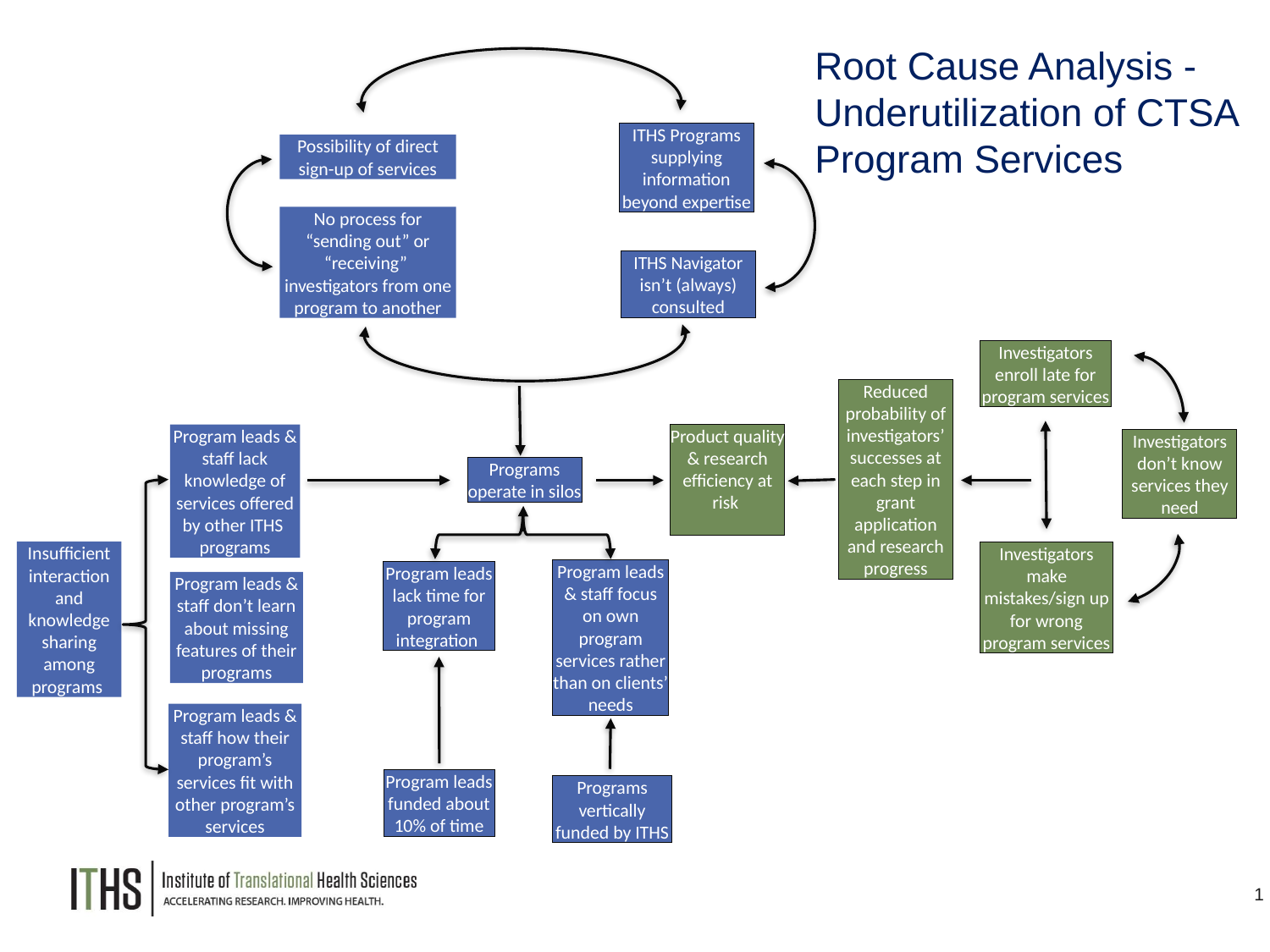

# Root Cause Analysis -Underutilization of CTSA Program Services
ITHS Programs supplying information beyond expertise
Possibility of direct sign-up of services
No process for “sending out” or “receiving” investigators from one program to another
ITHS Navigator isn’t (always) consulted
Investigators enroll late for program services
Reduced probability of investigators’ successes at each step in grant application and research progress
Product quality & research efficiency at risk
Program leads & staff lack knowledge of services offered by other ITHS programs
Investigators don’t know services they need
Programs operate in silos
Insufficient interaction and knowledge sharing among programs
Investigators make mistakes/sign up for wrong program services
Program leads & staff focus on own program services rather than on clients’ needs
Program leads lack time for program integration
Program leads & staff don’t learn about missing features of their programs
Program leads & staff how their program’s services fit with other program’s services
Program leads funded about 10% of time
Programs vertically funded by ITHS
