# Supplementary material for: Exploratory study of the underutilization of CTSA module services
Source: J Clin Transl Sci. 2022 Aug 10;6(1):e114. doi: 10.1017/cts.2022.440 (PMC9549576; doi:10.1017/cts.2022.440)
Supplement: Supplementary file 1 [file S205986612200440Xsup001.zip › S205986612200440Xsup003.docx]

**2020 Interview Protocols for Those Using Services in a Single CTSA Program and for Using Services in more than one CTSA Program**

**Interview background, email invitation and questions for investigators who have used services from only one CTSA program**

1. Background

Interview questions for investigators who, between January 2016 and December 2018, have used services in two or more programs at our CTSAs. The goal of the interview is to glean their experiences from those who used services from only one program at our CTSA. (We’re omitting REDCap use.)

It will be helpful to tape the interview and to have it transcribed. Feel free to add more space between each written question to add your own notes.

Before each interview, fill-in Q5 with services each interviewee used your across two or more of your CTSA’s programs. I don’t think it is critical to read to the respondent all the services they used, particularly if they have used a lot of services in one or more programs.

1. Email to schedule interviews

Subject: Services from [CTSA]

My name is [your name] and I am [your title] at the [CTSA] at [institution]. I am conducting interviews with a sample of professionals who have used [CTSA] services between January 2016 and December 2018. The purpose of the interview is to explore whether [CTSA] is meeting our service goals in a timely fashion. Your name was selected among a group that have used one or more [CTSA] services during this period. The interview will take about 60 minutes. Your participation in the interview is strictly voluntary. I am very grateful for your time and assistance with helping us improve services offered by [CTSA].

Please indicate each 60 minute period of those times listed below that would be convenient for you to participate.

- Any 60 minute period on [date 1]
- Any 60 minute period on [date 2]

If you have any questions or there are other times for an interview that work better for you, please send them to me, [name], [title], [CTSA] at [email address] or [phone].

Thank you,

[name]

[signature block]

1. Add to calendar

**Date of interview: ______________________________________**

**Time of interview: ______________________________________**

**Name of CTSA User to be interviewed (interviewee): _________________________________________**

1. Interview questions for investigators who have used services from only one [CTSA] program

**Introduction:** The [CTSA] has provided services to medical researchers for about 12 years. At [CTSA] we’re interested in understanding the way our services are used and we’re interviewing some investigators who have used services from only one [CTSA] program between January 2016 and December 2018. The interview is voluntary. It is neither confidential nor anonymous, but I won’t be divulging your identity unless it is important, for example, there is something that our PI needs to follow-up on. Do I have your permission to record this interview? I’ll use the recording to augment my notes.

Q1. When was the last time you remember using an [CTSA] service?

Q1a. What service or services did you use then?

It looks like you used….

|  | **Begin date of service** | **End date of service** | **Name of CTSA Service** | **(Their) Project Name** | **CTSA**  **Program** | **Resp. knew** |
| --- | --- | --- | --- | --- | --- | --- |
| 1 |  |  |  |  |  |  |
| 2 |  |  |  |  |  |  |
| 3 |  |  |  |  |  |  |

Q1b. How did that go for you?

Q2. Did you know this service was from [CTSA]?

Q3. What prompted you to pursue [CTSA] services?

Q4. I’d like to take a step back and ask how you found out about [CTSA] services.

Q3a. Were there multiple sources of information about [CTSA] services or just one?

Q3b. Who was that source (or were those sources)?

Q5.  Do you think you would use this service again?

(If “No”) Q5a. Would you describe why do you don’t think you’ll use this service again? (Follow-up: Do you have some ideas about changes we could make that would make it easier for you to use (CTSA name) service?

Q6. Did anyone follow-up with you about other services you might need? (E.g. writing a grant, sample selection, statistical help, analysis, publication)

Q7. Did you experience any barriers to using [CTSA] services?

Q7a. Please describe those barriers.

Q8. Do you think your colleagues would benefit from any of these services?

Q9. Are there services you wish your CTSA provided?

Q10. Do you have any comments you would like me to add to my notes?

Q11. Do you have any questions for me?

**Closing:** Thank you for your time. Your answers will help us improve [CTSA] services for all our users. Good bye. (Turn off recorder.)

1. Wrap up
   1. Add your notes
   2. Save recording to drive
   3. Send recording to transcriber
   4. Update records on status of interview

**Interview background, email invitation and questions for investigators who have used services from two or more CTSA programs**

1. Background

Interview questions for investigators who, between January 2016 and December 2018, have used services in only one program at our CTSAs. The goal of the interview is to glean their experiences from those who have used services in two or more programs at our CTSAs. (We’re omitting REDCap use.)

It will be helpful to tape the interview and to have it transcribed. Feel free to add more space between each written question to add your own notes.

Before each interview, fill-in Q1a with the single program services used by the interviewee. I don’t think it is critical to read to the respondent all the services they used, particularly if they have used a lot of services in one or more programs.

B. Email to schedule interviews (see above)

C. Add to calendar

**Date of interview: ______________________________________**

**Time of interview: ______________________________________**

**Name of CTSA User to be interviewed (interviewee): _________________________________________**

**Introduction:** The [CTSA] has provided services to medical researchers for about 12 years. At [CTSA] we’re interested in understanding the way our services are used and we’re interviewing some investigators who have used two or more [CTSA] services between January 2016 and December 2018. The interview is voluntary. It is neither confidential nor anonymous, but I won’t be divulging your identity unless it is important, for example, there is something that our PI needs to follow-up on. Do I have your permission to record this interview? I’ll use the recording to augment my notes.

Q1. When was the last time you remember using an [CTSA] service?

Q1a. What service or services did you use then?

Q1b. How did that go for you?

Q2. What prompted you to pursue [CTSA] services?

Q3. I’d like to take a step back and ask how you found out about [CTSA] services.

Q3a. Were there multiple sources of information about [CTSA] services or just one?

Q3b. Who was that source (or were those sources)?

Q4. What are the other services have you used from [CTSA]? (Check match in answers to Q5)

Q5. It looks like you used ……

|  | **Begin date of service** | **End date of service** | **Name of CTSA Service** | **(Their) Project Name** | **CTSA**  **Program** | **Resp. knew** |
| --- | --- | --- | --- | --- | --- | --- |
| 1 |  |  |  |  |  |  |
| 2 |  |  |  |  |  |  |
| 3 |  |  |  |  |  |  |
| 4 |  |  |  |  |  |  |
| 5 |  |  |  |  |  |  |
| 6 |  |  |  |  |  |  |

Q5a. Did you know these were services from [CTSA]?

Q5b. Did you come back to [CTSA] each time or was there someone who guided you to the next service?

Q6. Did anyone follow-up with you about other services you might need? (E.g. writing a grant, sample selection, statistical help, analysis, publication)

Q7. Do you think you would use any of those services again?

(If “No”) Q7a. Would you describe why do you don’t think you’ll use [CTSA] services again? (Follow-up: Do you have some ideas about changes we could make that would make it easier for you to use [CTSA] services?)

Q8. Did you experience any barriers to using [CTSA] services?

Q8a. Please describe those barriers.

Q9. Do you think your colleagues would benefit from any of these services?

Q10. Are there services you wish your CTSA provided?

Q11. Do you have any comments you would like me to add to my notes?

Q12. Do you have any questions for me?

**Closing:** Thank you for your time. Your answers will help us improve [CTSA] services for all our users. Good bye. (Turn off recorder.)

1. Wrap up
   1. Add your notes
   2. Save recording to drive
   3. Send recording to transcriber
   4. Update records on status of interview
