# Supplementary material for: Exploratory study of the underutilization of CTSA module services
Source: J Clin Transl Sci. 2022 Aug 10;6(1):e114. doi: 10.1017/cts.2022.440 (PMC9549576; doi:10.1017/cts.2022.440)
Supplement: Supplementary file 1 [file S205986612200440Xsup001.zip › S205986612200440Xsup005.docx]

**Modules Covered in CTSA (✔) by Number of Modules Managers Completing Survey (Some respondents reported on more than one module)**

| **Included Modules** | **Small**  **CTSA**  **(# of respondents)** | **Medium CTSA**  **(# of respondents)** | **Large**  **CTSA**  **(# of respondents)** | |
| --- | --- | --- | --- | --- |
| Biomedical Informatics | ✔ (0) | ✔ (0) | ✔ (1) | |
| Community Engagement | ✔ (1) | ✔ (2) | ✔ (2) | |
| Translational Workforce Development | ✔ (2) | ✔ (1) | ✔ (2) | |
| Biostatics, Epidemiology & Research Design | ✔ (1) | ✔ (1) | ✔ (2) | |
| Participant & Clinical Interactions | ✔ (3) | ✔ (1) | ✔ (3) | |
| Regulatory Knowledge & Support | ✔ (1) | ✔ (1) | ✔ (4) | |
| Integrating Special Populations | ✔ (1) | ✔ (1) | ✔ (1) | |
| Liaison to Trial Innovation Centers | ✔ (1) | -^a^ | ✔ (5) | |
| Liaison to Recruitment Innovation Centers | ✔ (1) | -^a^ | ✔ (3) | |
| Gene and Cell Therapy Lab |  |  | ✔ (2) | |
| **Excluded Programs** |  |  |  | |
| Team Science^b^ | ✔ | -^a^ | ✔ | |
| Pilots^c^ | ✔ | ✔ | ✔ | |
| KL2^b^ | ✔ | ✔ | ✔ | |
| TL1^b^ | ✔ | ✔ | ✔ | |
| ^a^ The Medium CTSA includes Liaison to Trial Innovation Centers and Liaison to Recruitment Innovation Centers as part of their navigator program and Team Science is folded into their education program.  ^b^ Team Science was mistakenly omitted from the program list.  ^c^ The Pilot, KL2 and TL1 programs provide research and training funding to successful applicants, and do not supply services to the larger clinical and translational research community. | | | |  |
